# Hybrid organic-inorganic polariton laser

**DOI:** 10.1038/s41598-017-11726-8

**Published:** 2017-09-12

**Authors:** G. G. Paschos, N. Somaschi, S. I. Tsintzos, D. Coles, J. L. Bricks, Z. Hatzopoulos, D. G. Lidzey, P. G. Lagoudakis, P. G. Savvidis

**Affiliations:** 1 FORTH, Institute of Electronic Structure and Laser, 71110 Heraklion, Crete Greece; 2 Department of Materials Science and Technology, University of Crete, 71003 Heraklion, Crete Greece; 3 School of Physics and Astronomy, University of Southampton, Southampton, SO17 1BJ United Kingdom; 4 Department of Physics and Astronomy, University of Sheffield, Sheffield, S3 7RH United Kingdom; 5 Institute of Organic Chemistry, National Academy of Sciences of Ukraine, Murmanskayaul. 5, Kiev, 02094 Ukraine; 6 Skolkovo Institute of Science and Technology, Novaya St., 100, Skolkovo, 143025 Russian Federation; 7 ITMO University, 197101 St. Petersburg, Russian Federation

## Abstract

Organic materials exhibit exceptional room temperature light emitting characteristics and enormous exciton oscillator strength, however, their low charge carrier mobility prevent their use in high-performance applications such as electrically pumped lasers. In this context, ultralow threshold polariton lasers, whose operation relies on Bose-Einstein condensation of polaritons – part-light part-matter quasiparticles, are highly advantageous since the requirement for high carrier injection no longer holds. Polariton lasers have been successfully implemented using inorganic materials owing to their excellent electrical properties, however, in most cases their relatively small exciton binding energies limit their operation temperature. It has been suggested that combining organic and inorganic semiconductors in a hybrid microcavity, exploiting resonant interactions between these materials would permit to dramatically enhance optical nonlinearities and operation temperature. Here, we obtain cavity mediated hybridization of GaAs and J-aggregate excitons in the strong coupling regime under electrical injection of carriers as well as polariton lasing up to 200 K under non-resonant optical pumping. Our demonstration paves the way towards realization of hybrid organic-inorganic microcavities which utilise the organic component for sustaining high temperature polariton condensation and efficient electrical injection through inorganic structure.

## Introduction

The ease of creation and manipulation of polariton Bose-Einstein condensates^1–5^ through direct optical pumping of semiconductor microcavities has recently led to demonstrations of numerous prototype polaritonic devices including low threshold lasers^6–9^, interferometers^10^ and transistor devices^11^. The later are almost entirely based on GaAs semiconductors that offer superior material quality, despite the restriction to low temperature operation due to the small exciton binding energy in this material^12, 13^. Organic microcavities owing to their very robust excitons^14^ are much more resilient and have been shown to support room temperature operation^15–17^. Furthermore, recent studies highlight their superfluid properties and long range propagation with potential use in interferometric devices^18, 19^.

In the pioneering work by Agranovich^20^, and co-workers it was shown that hybrid organic-inorganic structures, which mix two degenerate excitonic species can combine many desirable properties such as large exciton Bohr radius, favouring polariton-polariton interaction and relaxation as well as large oscillator strength for room temperature operation. Furthermore, optical nonlinearities in such systems were predicted to be increased by up to two orders of magnitude due to hybridization of the excitonic species^21^. Recent observations of the strong light-matter coupling in such hybrid structures^22, 23^, highlights their potential, albeit no observation of optical nonlinearities or lasing have been reported up to now.

**Figure 1:**
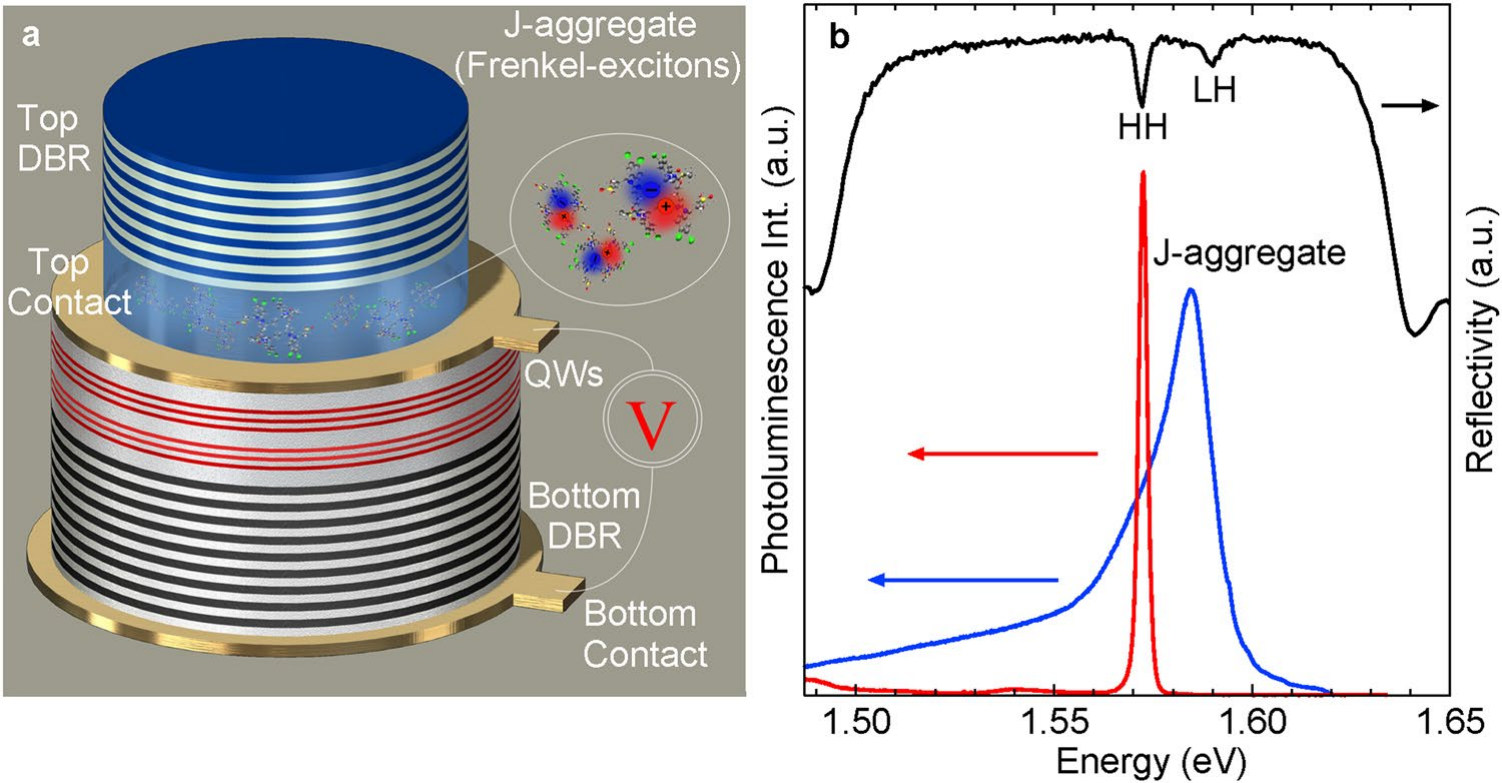
Hybrid microcavity structure with bottom distributed Bragg reflector (DBR) reflectivity and emission spectra. (**a**) Full schematic representation of the hybrid microcavity LED incorporating, bottom DBR mirror, inorganic QWs, spin coated J-aggregate layer, electrical contacts and the top dielectric DBR mirror. (**b**) Reflectivity and photoluminescence spectra at 25 K from only inorganic half microcavity. Organic J-agg film photoluminescence at same temperature.

Here, we obtain cavity photon-mediated hybridization of Frenkel and Wannier-Mott excitons in a hybrid microcavity formed by incorporation of organic J-aggregate dye and inorganic GaAs quantum wells (QWs) inside a high quality factor optical cavity (Q~2000). The constituents of the hybrid microcavities are described under Materials and Methods. The resulting mixed bosonic polariton quasiparticles, owing to their hybrid nature are shown to exhibit lasing under pulsed optical excitation that persists up to 200 K. Furthermore, electrical contacts (see Fig. 1a) defined on to the inorganic part of the device allow for efficient electrical injection of the whole system via ultrafast energy exchange between different excitonic species in a mixed polariton state.

## Results

### Hybrid polaritons

**Figure 2:**
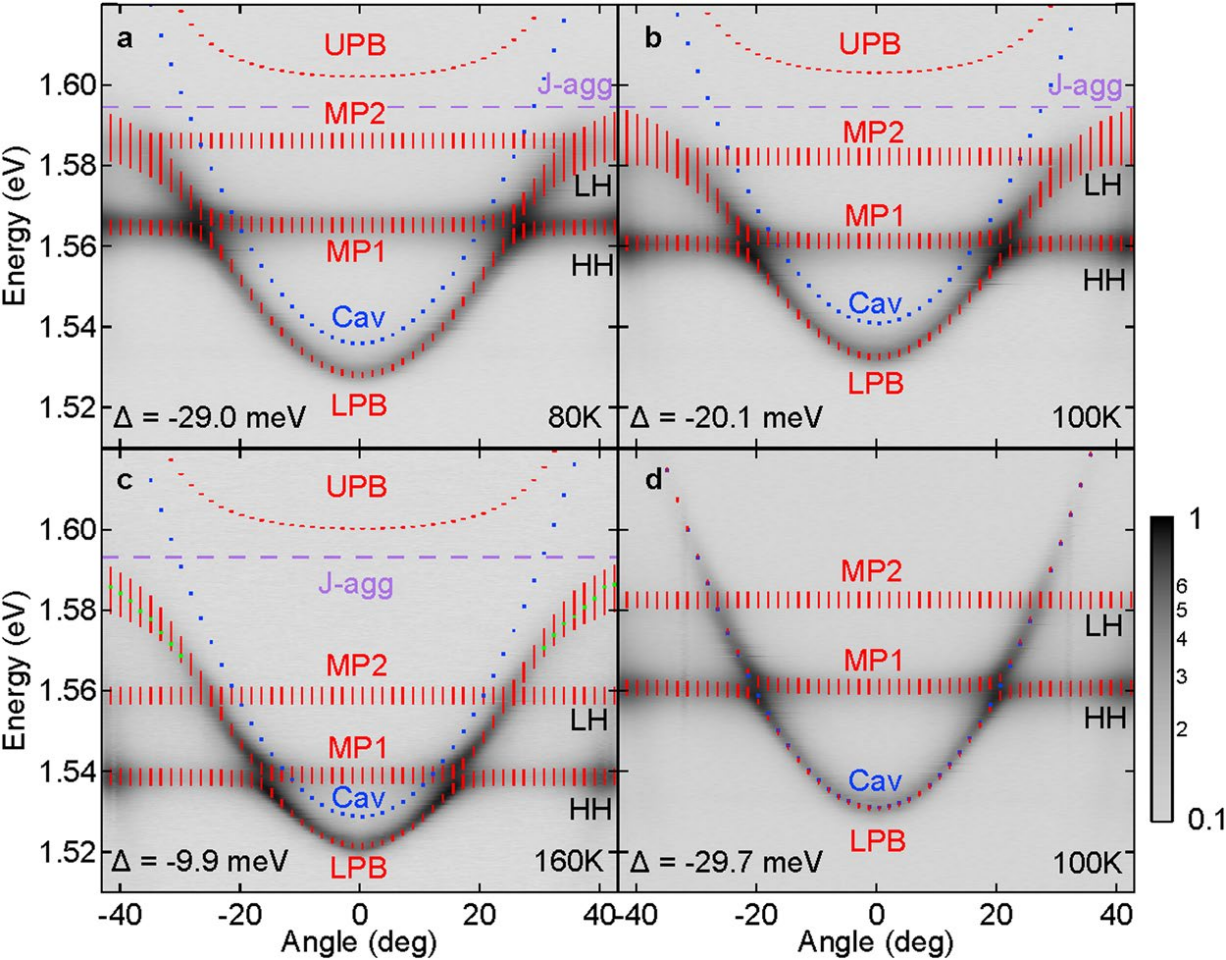
Far-field normalized PL emission images for different temperatures and detunings. (**a**) Δ = −29.0 meV at 80 K, (**b**) Δ = −20.1 meV at 100 K and (**c**) Δ = −9.9 meV at 160 K showing emission from the bottom (LPB) and middle (MPB) polariton branches. Clear anticrossing between the heavy hole and J-aggregate excitons with cavity mode can be observed. A Rabi splitting (Ω) of ~50 meV is extracted from the fittings using four coupled oscillator model. The green dots represent extracted peak positions from the corresponding spectra (see Supplementary Fig. S1) and the blue dots indicate the bare cavity mode. (**d**) Microcavity dispersions in the absence of J-aggregate at 100 K showing bare cavity mode parabolic dispersion. Contour plot spectral profiles at different angles are presented in Supplementary Fig. S8.

We study the linear properties of hybrid exciton-polaritons in using 0.5 mW of continuous wave optical excitation tuned above the stopband of the dielectric mirror at 1.908 eV. The sample is held in a closed cycle-cryostat at elevated temperatures ranging from 80–160 K in order to obtain sufficiently strong photoluminescence (PL) at the wavevectors of the expected anti-crossing between the cavity and exciton modes. The measurements are performed with the use of a linear polarizer, recording only the TE polarization for clarity. Figure 2(a–c) shows the dispersion for different temperatures and detunings (Δ); Δ here is defined as the energy difference between the QW heavy hole (HH) exciton and the cavity mode (CM). By increasing the temperature, we observe a redshift of the GaAs exciton energy as it is clearly seen by the change of the HH exciton energy between Fig. 2(a–c), while the J-aggregate exciton energy varies much less with temperature in agreement with previous observations^24^.

The normalized photoluminescence from the lower polariton (LPB) and middle polariton (MPB) branches of Fig. 2(a–c) is superimposed with the calculated hybrid polariton dispersion of the LPB, MPB and upper polariton branch (UPB). The modeled dispersions are calculated using a four coupled oscillator model that includes the HH and light hole (LH) excitons of the GaAs QWs, the J-aggregate excitons and the cavity photon mode^25–27^ with corresponding linewidth broadenings. The J-aggregate exciton linewidth is γ_J-agg_ = 13 meV at 80 K and remains nearly constant for the whole experimental temperature range. The corresponding cavity mode linewidth is γ_cav_ = 0.1 meV while HH and LH excitons linewidths vary slightly with temperature from γ_HH_ = 3.6 meV, γ_LH_ = 3.7 meV at 80 K to 4.2 meV, 4.5 meV at 160 K respectively (see Supplementary Table S3). In this simple coupled oscillators model, we have neglected non-radiative resonance energy transfer due to the large inorganic-organic spatial separation. The blue dotted line refers to the bare cavity mode energy, while the red dashed lines mark the full width at half maximum (FWHM) of the corresponding polariton branches. From the fitted dispersion curves we obtain a Rabi splitting of 4.5 meV for the GaAs HH exciton and 50 meV for the larger oscillator strength J-aggregate excitons respectively. The corresponding polariton linewidth at the crossing point of cavity mode with J-aggregate is ~6.5 meV.

The non-uniform distribution of J-aggregates in the suspension matrix, allows us to locate areas of the sample with negligible concentrations of J-aggregate. Figure 2d shows the dispersion of the PL in the absence of strong coupling with J-aggregate while the system is strongly coupled to only GaAs excitons. We note the difference in dispersions presented in Fig. 2c, d around ~1.58 eV. The green dots in Fig. 2c (see Supplementary Fig. S1) show a clear deviation between the parabolic dispersion of bare cavity mode (blue dots), also shown in Fig. 2d, and the LPB of Fig. 2c, providing unequivocal evidence of strong coupling with the J-aggregate in Fig. 2(a–c).

The Hopfield coefficients between the cavity, J-aggregate, HH and LH excitons used to fit the experimental dispersion of the LPB of Fig. 2b show that k_||_ = 0, exhibit substantial mixing of all four components (0.88, 0.075, 0.04, 0.005 respectively, also shown in Supplementary Fig. S2). In particular, Frenkel and cavity photon components dominate the polariton character at normal incidence, while the Wannier-Mott excitons contribution becomes considerable at large angles.

An important implication for the efficiency of the pumping scheme in the studied structure is that GaAs QW with exciton energy lying below J-aggregate exciton energy serves as efficient reservoir for accumulation of injected carriers. These subsequently feed lower polariton states near the HH anticrossing point with the lower polariton branch as seen from the bright emission in Fig. 2(a–c).

### Hybrid polariton nonlinearities

**Figure 3:**
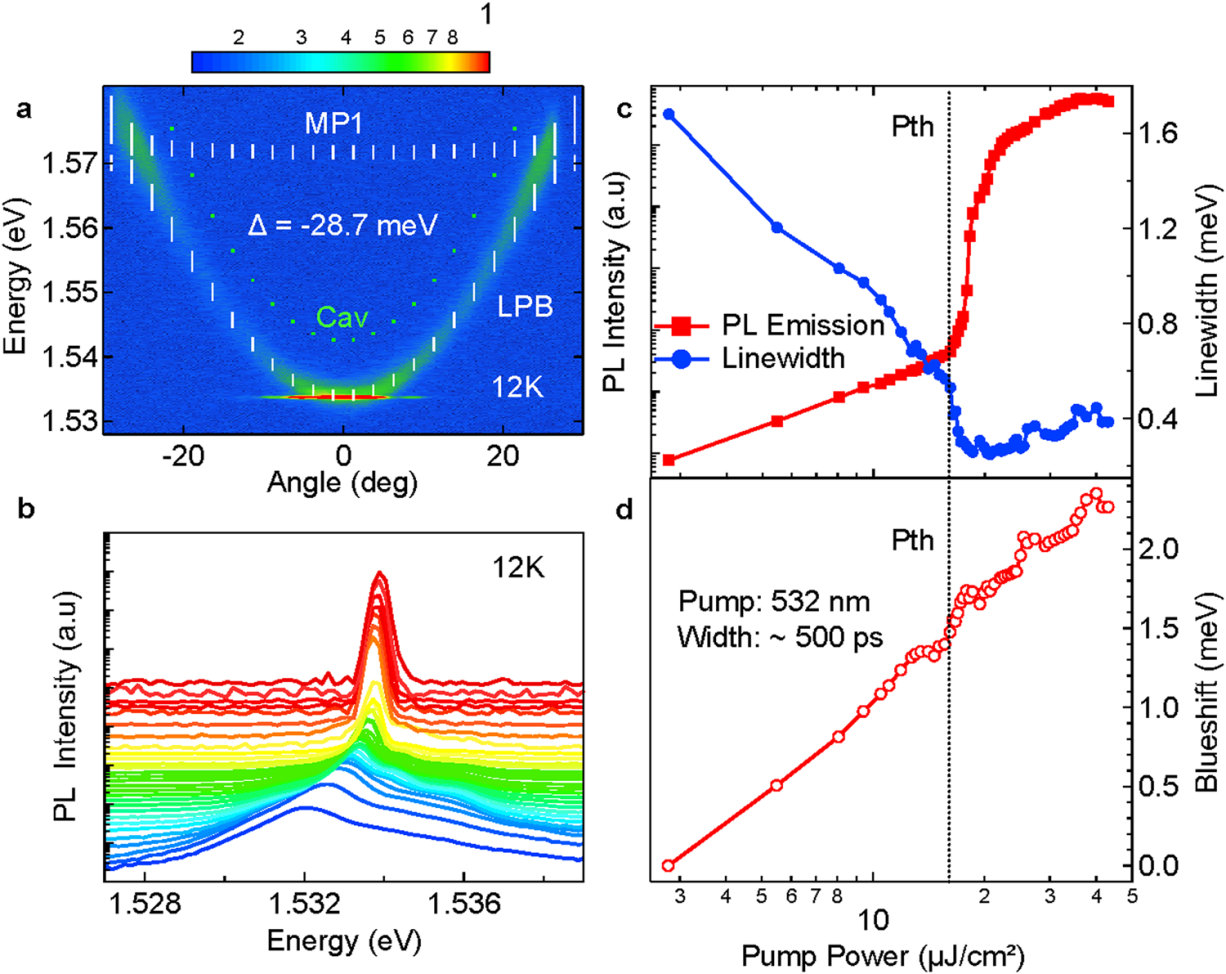
Hybrid polariton lasing. (**a**) Angle resolved far-field normalized emission of the onset of lasing at 12 K and sample detuning value of Δ = −28.7 meV. (**b**) Normal incidence PL intensity vs pump power. (**c**) Integrated PL intensity and linewidth with increasing pump power of the LPB. Corresponding threshold (Pth) of 16.1 µJ/cm^2^ marked by dashed line. (**d**) LPB energy blueshift vs excitation power.

Nonlinear properties of hybrid polaritons are studied by optically exciting the structure with quasi-CW YAG laser at 532 nm with 500 ps pulse duration and a repetition rate of 7.58 kHz. The diameter of the excitation spot size is 5 μm. In Fig. 3a we provide evidence of hybrid polariton lasing by the narrow and intense emission at normal incidence above threshold. Fitted polariton branch dispersions show that lasing emission occurs at least 10 meV below the corresponding bare cavity mode dispersion confirming that the observed nonlinearity occurs indeed in the strong coupling regime with J-aggregate excitons. Furthermore, our theoretical gain calculations presented in supplementary Fig. S4, show that at the injected carrier densities used in the experiment, no population inversion and gain can be supported by the GaAs QW active medium at the lasing energy and highlight the role of hybridization in achieving polariton lasing in this device.

Normal incidence PL spectra with increasing excitation power is plotted in Fig. 3b. It shows nonlinear increase in the PL intensity accompanied by continuous blueshift (see also Supplementary Mov. S5) of the lower polariton branch which originates from both J-aggregate and QW exciton-exciton interactions^28^. Such interactions are considerably weaker for the tightly bound Frenkel excitons characteristic of organic semiconductors, therefore largest contribution to the blueshift arises from GaAs QW excitons. Assuming characteristic exciton-exciton interaction constant of ~6.5 μeV·μm^2^ for such QWs and energy blueshift dependence on carrier density ΔΕ = g N, we estimate that the carrier density at the threshold when ΔΕ = 1.48 meV per QW is ~3.78 10^9^ cm^−2^, well below Mott density. The corresponding integrated emission together with the FWHM emission linewidth as well as blueshift are plotted in Fig. 3(c,d) respectively. Expectedly, nonlinear increase in the emission is accompanied by significant line narrowing of the PL emission around threshold as shown in Fig. 3c at 16.1 μJ/cm^2^. At higher excitation powers, the emission is broadened slightly due to increased scattering from and to the exciton reservoir and within the condensate itself.

Identical power dependent PL measurements using stretched regenerative amplifier pulses of ~3 ps duration, reveal similar lower polariton energy blueshift at the corresponding threshold value of 13.9 µJ/cm^2^ confirming that the onset of lasing regime occurs indeed at similar carrier densities despite very different excitation conditions used (see Supplementary Fig. S6). This is because for a typical carrier lifetime in the order of 1ns^29^, both excitation schemes use pulse durations ≤500 ps, which are sufficiently small to ignore any recombination effects, resulting in the same maximum carrier concentrations created at the end of the pulse. However, discrepancies are observed above threshold in the overall blueshift, for the two cases. Notably, the high repetition rate laser ~250 kHz delivers larger average power onto the sample causing sample heating and energy redshift which counteracts the expected energy blueshift from the rising power density^30, 31^. In contrast, low repetition rate laser produces minimal heating and blueshift continues up to highest recorded excitation powers. Furthermore, high repetition rate laser and subsequent heating induce photobleaching^32^ of the sample above threshold, as opposed to very robust lasing operation under low repetition rate excitation.

**Figure 4:**
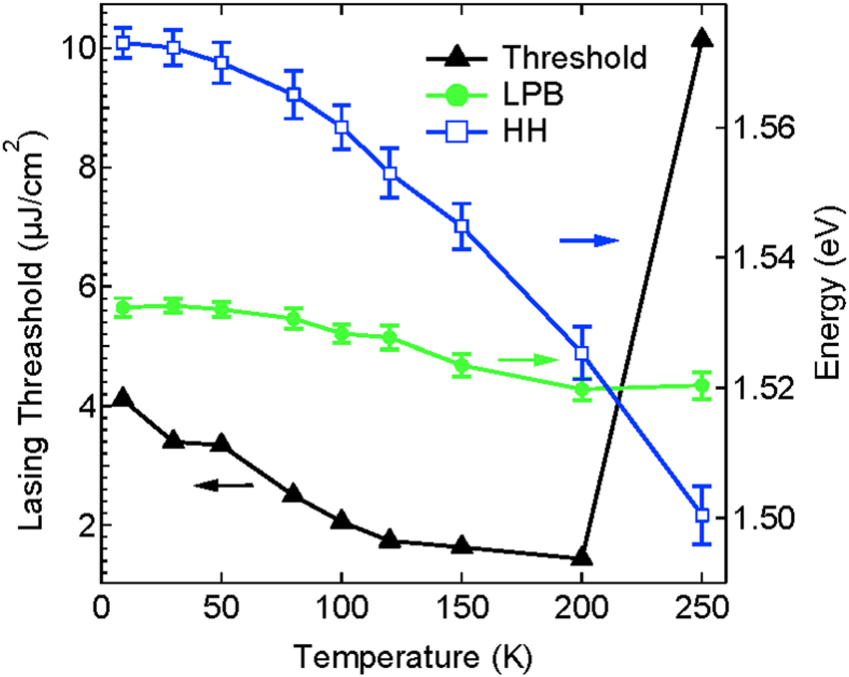
Lasing threshold. Temperature dependence of polariton lasing threshold of hybrid device. Lasing threshold decreases gradually with temperature until the HH exciton reservoir energy redshifts below LPB marked by a sharp increase in the threshold (250 K).

Efficient population of lower energy polariton states responsible for the onset of lasing is achieved by the presence of HH exciton reservoir in the vicinity of the LPB capable of directly feeding lower polariton states below relaxation bottleneck. We therefore study the effect of temperature and energy position of the feeding reservoir on the lasing threshold of our hybrid device. Strikingly, for a negatively detuned Δ = −25.2 meV device, the lasing threshold gradually decreases with increasing temperature from 9 K up to 200 K as seen in the Fig. 4. This threshold reduction is obtained by feeding polaritons closer to the lowest lying polariton states requiring shorter relaxation paths. The lasing threshold increases sharply when HH exciton energy approaches the lower polariton branch due to the rapid change in the LPB density of states^33^ as well as inability of HH exciton reservoir to efficiently populate LPB states once its energy is below cavity mode.

We note that identical structures without J-aggregate dye show no polariton lasing at such large negative detunings. Furthermore, these structures do not exhibit lasing even in the weak coupling regime, since gain arising from population inversion has no overlap with the cavity mode.

### Hybrid polariton LED

**Figure 5:**
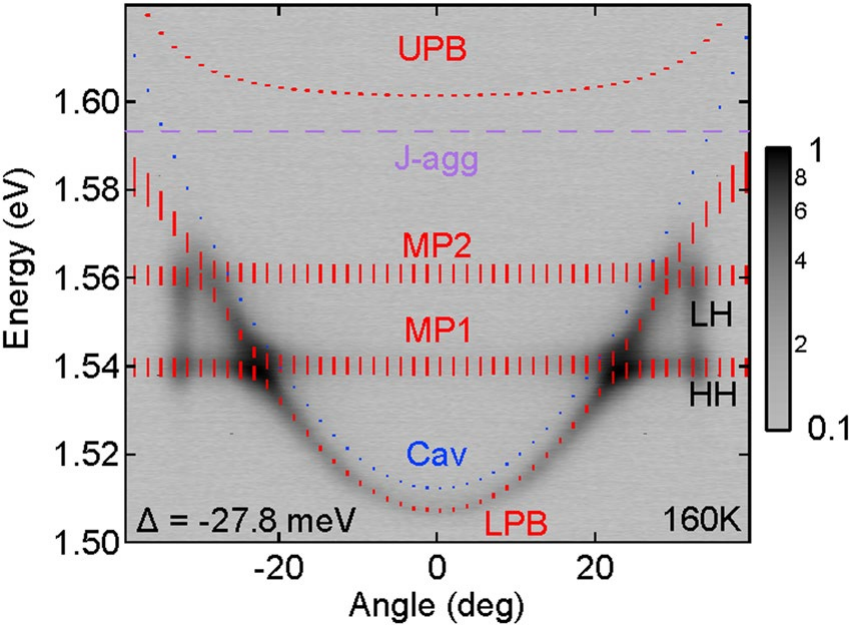
Normalized EL emission from hybrid states fed by HH, LH GaAs exciton reservoirs. Far field electroluminescence image taken at detuning of Δ = −27.8 meV at 160 K, showing hybrid polariton emission under forward DC bias. Fitted polariton dispersion branches calculated using four coupled harmonic oscillator model. Blue dotted line represents bare cavity mode dispersion. Contour plot spectral profiles at different angles are presented in Supplementary Fig. S8.

We exploit the hybrid nature of exciton polariton states to efficiently exchange energy between inorganic and organic excitations and build a prototype hybrid polariton light emitting device described in detail in the materials and methods section. Electrical contacts are fabricated onto the inorganic section of the device allowing for direct intracavity injection of carriers into the GaAs QWs. A mesa with 350 μm outer diameter and 150 μm inner diameter is operated under forward DC bias producing electrical current of 1.61 mA. Due to strong mixing of polariton states the injected excitons are rapidly transferred^34^ from the inorganic excitons to the organic counterparts radiatively, resulting in strong electroluminescence (EL) presented in Fig. 5. The device shows characteristic anticrossing behaviour and operates in the strong coupling regime even at elevated temperatures of 160 K. However, in the present device only small fraction of injected carriers are captured inside QWs with the escaping carriers contributing strongly to EL emission arising from the DBR barriers. By comparing LPB blueshits observed in EL measurements under highest possible electrical injection current of 5.01 mA, with PL measurements, we conclude that, similar blueshift of 0.8 meV is produced under optical excitation with 4μJ/cm^2^, which is ~4 times below lasing threshold. Therefore, we believe that further improvements of the electrical contacts in the presence of organics as well as better carrier capture efficiency will be necessary to achieve lasing operation under electrical injection.

## Discussion

The results presented here, show that it is possible to achieve efficient energy exchange between inorganic and organic exciton species via their hybridization with the cavity photon in a polariton state. We exploit such resonant interactions and the hybrid nature of polaritons to dramatically enhance optical nonlinearities in our system allowing demonstration of polariton lasing at 200 K. Furthermore, the advantage of the implemented electrical injection scheme is that it does not require the use of low mobility organic hole injection layers that typically limits device performance^35^. Instead, the present device relies on well-established GaAs technology to electrically inject carriers into inorganic part of the device^36^ and uses strong coupling regime to efficiently transfer excitation from inorganic to organic semiconductor excitations, thus promising a new route to electrical injection of organic active medium.

## Methods

### Sample preparation

The microcavity sample investigated in the present work is an organic-inorganic hybrid microcavity light emitting diode - LED, depicted in Fig. 1a. The structure consists of an active layer, composed of III-V semiconductor quantum wells and a molecular J-aggregate forming cyanine dye film, embedded in between two distributed Bragg reflectors (DBR) mirrors. The bottom DBR is formed by molecular beam epitaxy (MBE) and consists of 24 λ/4 pairs of n-type doped AlAs/GaAs layers, followed by six GaAs/AlGaAs quantum wells capped by a p-type AlGaAs layer which acts as a spacer at the interface with the organic film. In Fig. 1b reflectivity spectra on inorganic part of the structure reveals HH, LH exciton resonances as well as QW PL emission which overlaps with bare J-aggregate PL. To ensure electrical isolation 350 µm diameter mesas are fabricated using reactive ion etching. The cyanine J-aggregate dye chosen for the purpose is the U3(3-[(2Z)-5-chloro-2-[((3E)-3-[5-chloro-3-(3-triethylammonium-sulfonatopropyl)-1,3benzothiazol-3-ium-2-yl]methylene-2,5,5-trimethylcyclohex-1-en1-yl)methylene]-1,3-benzothiazol-3(2 H)-yl] propane-1-sulfonate)^24^ and its chemical structure is shown in Supplementary Fig. S7. After the U3 dye being previously dissolved in a water based poly(vinyl alcohol) matrix solution (3 mg/ml), the device is completed by spin casting organic J-aggregate U3 dye film of ~90 nm thickness followed by evaporation of the top dielectric mirror consisting of 8 alternating pairs of Ta_2_O_5_/SiO_2_ deposited in an oxygen plasma atmosphere.

## Electronic supplementary material

Supplementary Information

Supplementary Video
