# Author Correction: Hybrid organic-inorganic polariton laser

**DOI:** 10.1038/s41598-018-24599-2

**Published:** 2018-04-18

**Authors:** G. G. Paschos, N. Somaschi, S. I. Tsintzos, D. Coles, J. L. Bricks, Z. Hatzopoulos, D. G. Lidzey, P. G. Lagoudakis, P. G. Savvidis

**Affiliations:** 1 FORTH, Institute of Electronic Structure and Laser, 71110 Heraklion, Crete Greece; 2 Department of Materials Science and Technology, University of Crete, 71003 Heraklion, Crete Greece; 3 School of Physics and Astronomy, University of Southampton, Southampton, SO17 1BJ United Kingdom; 4 Department of Physics and Astronomy, University of Sheffield, Sheffield, S3 7RH United Kingdom; 5 Institute of Organic Chemistry, National Academy of Sciences of Ukraine, Murmanskayaul. 5, Kiev, 02094 Ukraine; 6 Skolkovo Institute of Science and Technology, Novaya St., 100, Skolkovo, 143025 Russian Federation; 7 ITMO University, 197101 St. Petersburg, Russian Federation

Correction to: *Scientific Reports* 10.1038/s41598-017-11726-8, published online 12 September 2017

This Article contains errors in References 18 and 19, where “Lerario, G.” is incorrectly listed as “Giovanni, L”. The correct References 18 and 19 appear below as Refs 1, 2 respectively.
