# Supplementary material for: Hybrid organic-inorganic polariton laser
Source: Sci Rep. 2017 Sep 12;7:11377. doi: 10.1038/s41598-017-11726-8 (PMC5595872; doi:10.1038/s41598-017-11726-8)
Supplement: Supplementary file 1 — Supplementary Information [file 41598_2017_11726_MOESM1_ESM.pdf]

Hybrid organic-inorganic polariton laser

G.G. Paschos[1,2*], N. Somaschi[1,3], S.I. Tsintzos[1], D. Coles[4], J.L. Bricks[5], Z. Hatzopoulos[1], D.G. Lidzey[4], P.G. Lagoudakis[3,6], P.G. Savvidis[1,2,7†]

[1]*FORTH, Institute of Electronic Structure and Laser, 71110 Heraklion, Crete, Greece*

[2]*Department of Materials Science and Technology, University of Crete 71003 Heraklion, Crete, Greece*

[3]*Department of Physics and Astronomy, University of Southampton, United Kingdom*

[4]*Department of Physics and Astronomy, University of Sheffield, United Kingdom*

[5]*Institute of Organic Chemistry, National Academy of Sciences of Ukraine, Murmanskayaul. 5, Kiev 02094, Ukraine*

[6]*Skolkovo Institute of Science and Technology Novaya St., 100, Skolkovo 143025, Russian Federation*

[7]*ITMO University, St. Petersburg 197101, Russian Federation*

***gianpaschos@materials.uoc.gr**, **†psav@materials.uoc.gr**

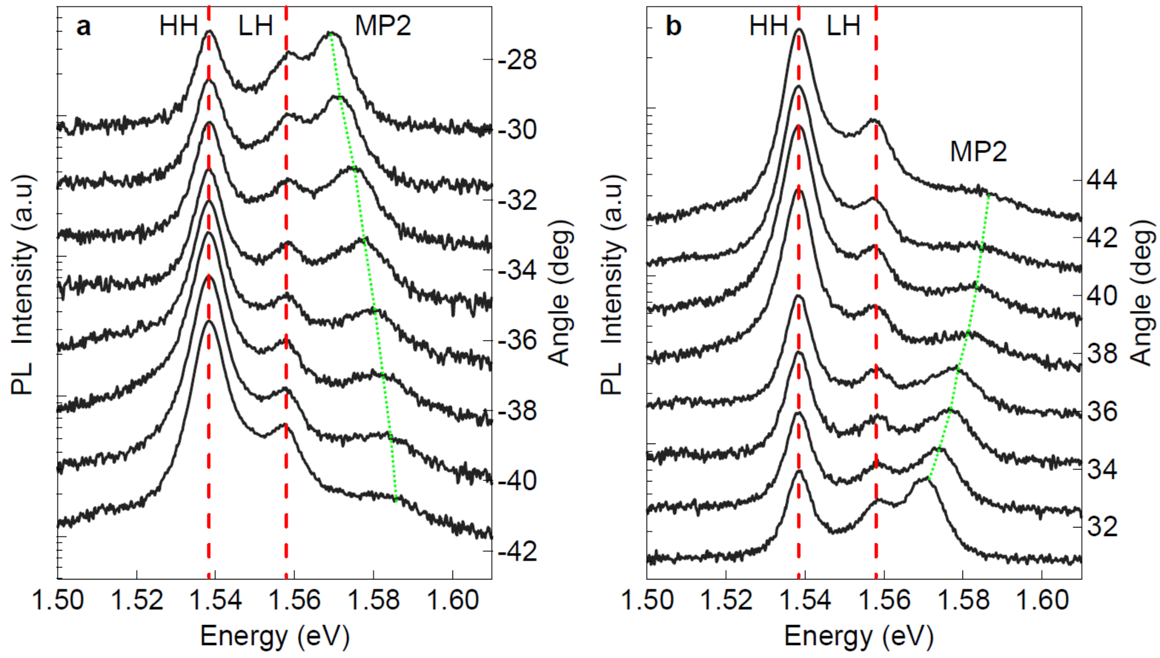

Fig. S1 | Polariton emission spectra from high angle PL measurements at 160K. a, Spectra at high negative angles. **b,** Spectra at high positive angles. The dashed lines denote the HH and LH GaAs excitons and the dotted lines the second middle polariton branch (MP2). The green dots in Fig.2c are extracted from the peak positions of the related MP2 spectra.

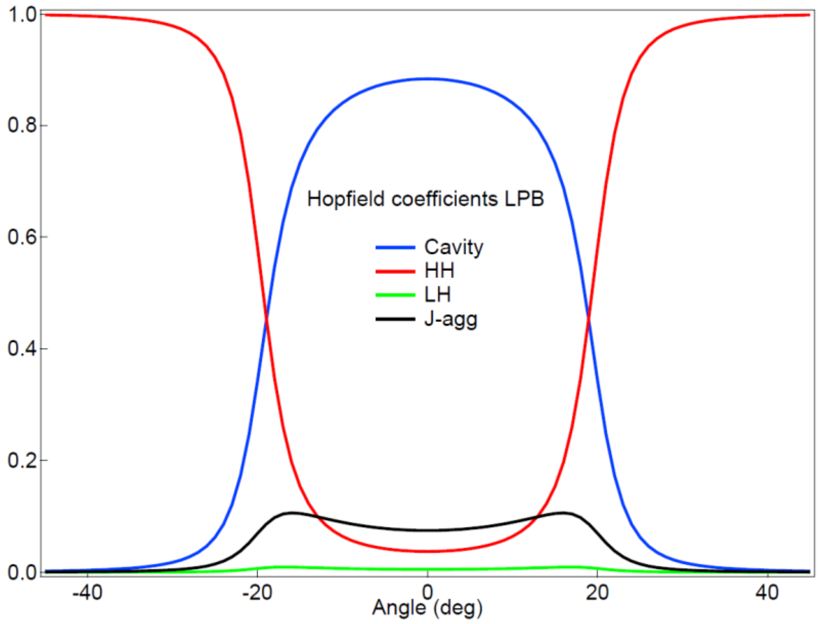

Fig. S2 | Hopfield coefficients of lower polariton branch. Hopfield coefficients of lower polariton branch (LPB) for experimental conditions reported in the Fig. 2b. At normal incidence the LPB possesses strong photonic character (0.88), while Frenkel exciton fraction, arising from the J-aggregate, has the next highest contribution with a value of 0.075. At higher angles, the LPB is dominated by HH component which is only 0.04 at normal incidence. Light hole exciton fraction in the hybrid state remains tiny throughout the angular range with its highest value at normal incidence of 0.005.

A four coupled harmonic oscillator model was used to calculate the relative cavity, heavy hole (HH), light hole (LH) and J-aggregate exciton fractions of the hybrid polariton state at each angle. The system can be described by the following matrix equation:

$$\begin{bmatrix} \boldsymbol{E_C(\theta) + i\gamma_c} & \frac{\hbar\Omega_1}{2} & \frac{\hbar\Omega_2}{2} & \frac{\hbar\Omega_3}{2} \\ \frac{\hbar\Omega_1}{2} & \boldsymbol{E_{HH} + i\gamma_{HH}} & \boldsymbol{0} & \boldsymbol{0} \\ \frac{\hbar\Omega_2}{2} & \boldsymbol{0} & \boldsymbol{E_{LH} + i\gamma_{LH}} & \boldsymbol{0} \\ \frac{\hbar\Omega_3}{2} & \boldsymbol{0} & \boldsymbol{0} & \boldsymbol{E_{J-agg} + i\gamma_{J-agg}} \end{bmatrix} \begin{bmatrix} \boldsymbol{C} \\ \boldsymbol{X_{HH}} \\ \boldsymbol{X_{LH}} \\ \boldsymbol{X_{J-agg}} \end{bmatrix} = \boldsymbol{E_p(\theta)} \begin{bmatrix} \boldsymbol{C} \\ \boldsymbol{X_{HH}} \\ \boldsymbol{X_{LH}} \\ \boldsymbol{X_{J-agg}} \end{bmatrix}$$

where, $E_C(\theta) = E_0\,(1 - (\sin\theta^2)/n^2)^{-1/2}$ is the cavity mode energy and γ_c is the related linewidth of the cavity mode. E_0 is the cavity resonance at normal incidence and n=1.8 is the intracavity effective refractive index which reflects mean value for dielectric and GaAs DBR mirror materials used to form the microcavity. E_{HH}, E_{LH}, E_{J-agg} and γ_{HH}, γ_{LH}, γ_{J-agg} are the respective energies and linewidths of the heavy hole, light hole and J-aggregate excitons respectively. In addition, $\frac{\hbar\Omega_1}{2}$ is the coupling between E_C and E_{HH}, $\frac{\hbar\Omega_2}{2}$ the coupling between E_C and E_{LH} and $\frac{\hbar\Omega_3}{2}$ the coupling between E_C and E_{J-agg}. The coefficients $|C|^2$, $|X_{HH}|^2$, $|X_{LH}|^2$ and $|X_{J-agg}|^2$, are the corresponding photonic and excitonic fractions of the hybrid polariton state at each angle. In this model no coupling between GaAs and J-agg excitons is taken into account due to large special separation between QWs and the organic film.

Table S3 | Fitting parameters for coupled oscillator model.

	n	E_0	Det	E_{HH}	E_{LH}	E_{J-agg}	γ_c	γ_{HH}	γ_{LH}	γ_{J-agg}	$\hbar\Omega_1$	$\hbar\Omega_2$	$\hbar\Omega_3$
Fig.2a	1.8	1.5359	-29	1.5649	1.5861	1.5945	0.1	3.6	3.7	13	6.8	2.6	50
Fig.2b	1.8	1.5409	-20.1	1.561	1.5822	1.5945	0.1	3.7	3.9	13	6.8	2.6	50
Fig.2c	1.8	1.5288	-9.9	1.5387	1.5588	1.5932	0.1	4.2	4.5	13	6.8	2.6	50
Fig.2d	1.8	1.5311	-29.7	1.561	1.5822	1.5945	0.1	3.7	3.9	13	6.8	2.6	50
Fig.5	1.8	1.5123	-27.8	1.5399	1.5608	1.5932	0.1	4.2	4.5	13	6.8	2.6	50

* All values are in meV

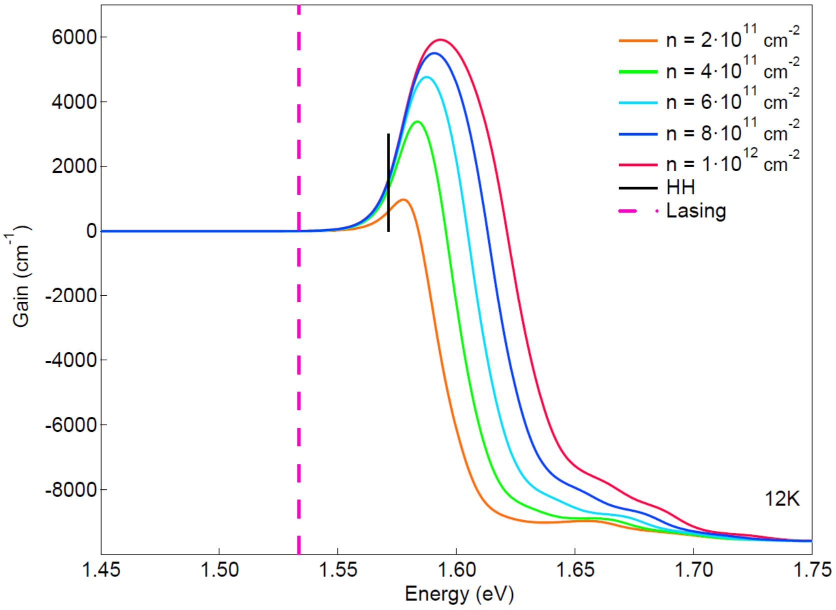

Fig. S4 | Gain calculations for different carrier density values. Theoretical gain calculations for a 7.5nm GaAs/$Al_{0.3}Ga_{0.7}As$ QW for different carrier densities shown in the inset, at 12K. In our calculations the valence band mixing effects are taken into account by solving the 4x4 effective-mass theory[1] (k · p method). Maximum gain is achieved for energies above exciton transition marked by solid line, whereas

corresponding values at lasing energy marked by dashed line are nearly zero confirming population inversion-less nature of the lasing process.

Mov. S5 | Video of power and angle-resolved PL. Video showing a series of far field emission images for increasing pump power. As pump power increases, a progressive reduction of the intensity coming from high wave-vectors is observed, while normal incidence lower polariton PL blueshifts due to polariton-polariton interaction and increases nonlinearly in intensity with simultaneous reduction in the emission linewidth.

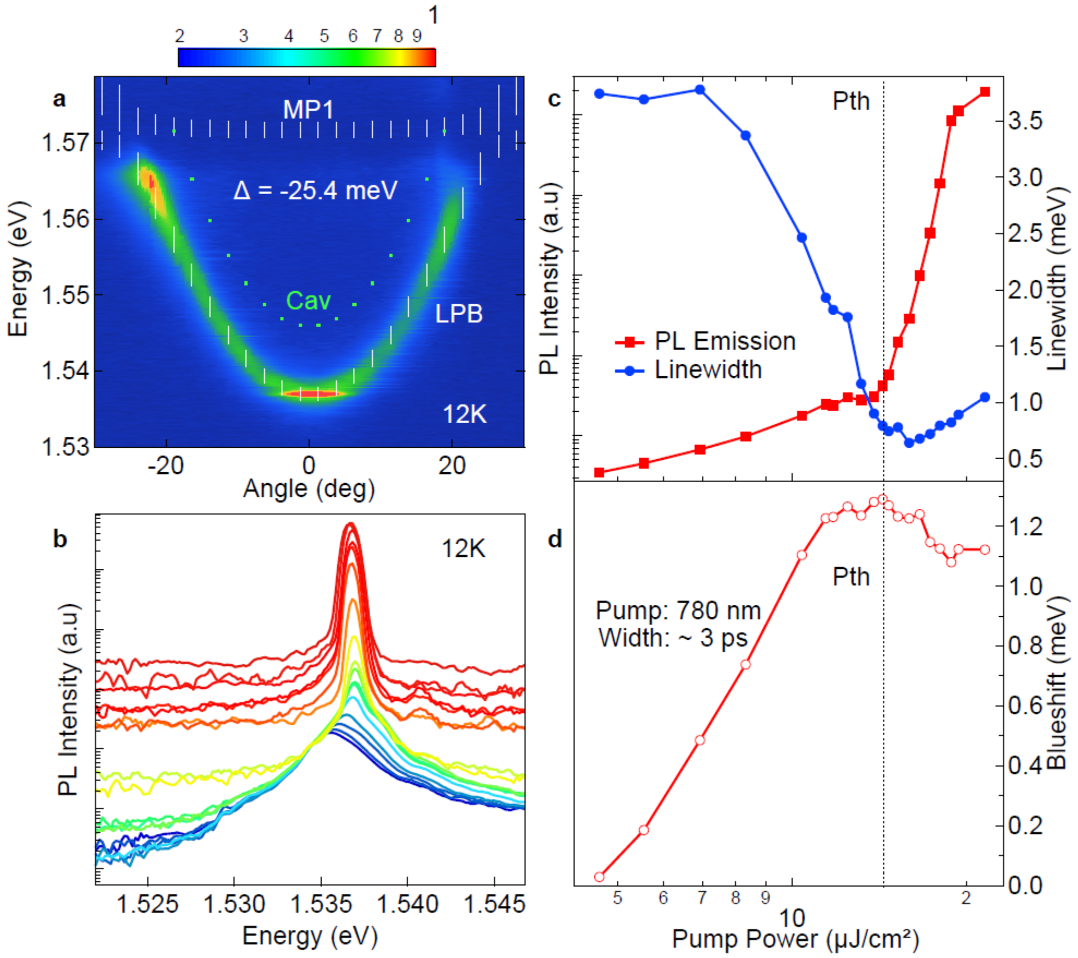

Fig. S6 | Power dependent PL with amplified short (~3 ps) pulse excitation. a, Angle resolved far-field normalized emission at the onset of polariton lasing at 12 K and sample detuning of Δ = -25.4 meV. **b,** Normal incidence PL intensity vs pump power. **c,** Integrated PL intensity and linewidth with increasing

pump power of the LPB. Corresponding threshold (Pth) of 13.9 μJ/cm^2 marked by dashed line. **d,** LPB energy blueshift vs excitation power.

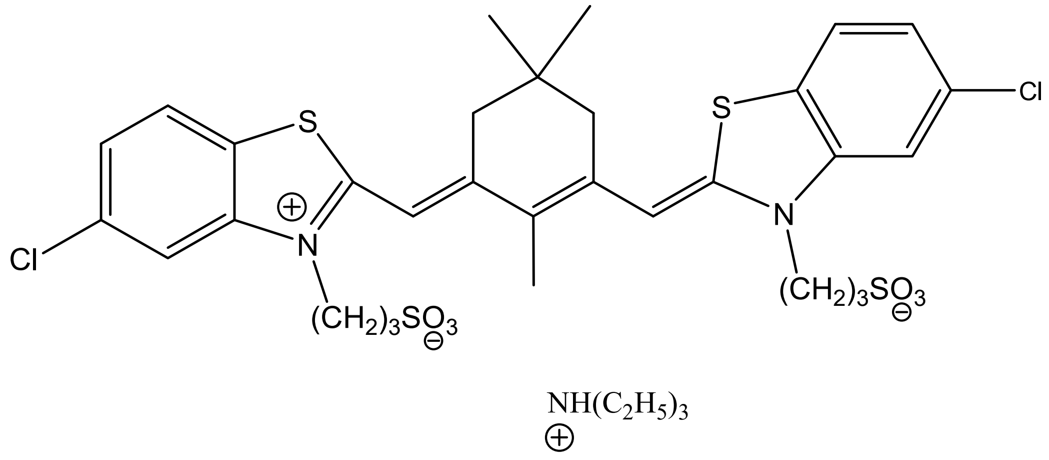

Fig. S7 | Chemical structure of U3 dye. Chemical structure of the synthesized U3 dye. The complete chemical formula of the U3 dye is $C_{37}H_{49}Cl_2N_3O_6S_4$.

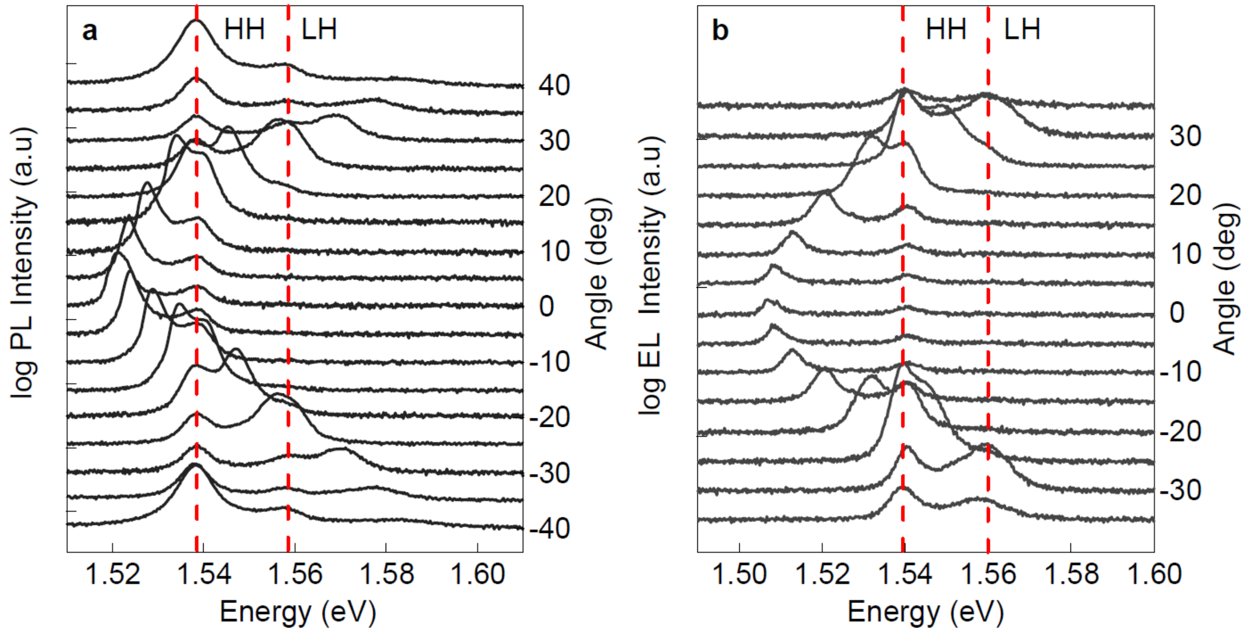

Fig. S8 | PL and EL measurements for various angles at 160K. a, Polariton PL emission for angles between -40º and 40º and temperature of 160K. **b,** Polariton EL emission for angles between -35º and 35º at 160K. The dashed lines denote the HH and LH GaAs exciton energy positions.

References:

1. Ahn, D. & Chuang, S.-L. Optical gain and gain suppression of quantum-well lasers with valence band mixing. *IEEE J. Quantum Electron.* **26,** 13-24 (1990).
